# Supplementary material for: Development of Novel ROCK Inhibitors via 3D-QSAR and Molecular Docking Studies: A Framework for Multi-Target Drug Design
Source: Pharmaceutics. 2024 Sep 26;16(10):1250. doi: 10.3390/pharmaceutics16101250 (PMC11514586; doi:10.3390/pharmaceutics16101250)
Supplement: Supplementary file 1 [file pharmaceutics-16-01250-s001.zip › pharmaceutics-3209615-supplementary.pdf]

pharmaceutics

MDPI

Article

Development of Novel ROCK Inhibitors via 3D-QSAR and Molecular Docking Studies: A Framework for Multi-Target Drug Design

Milan Beljkas [1], Milos Petkovic [2], Ana Vuletic [3], Ana Djuric [3], Juan Francisco Santibanez [4], Tatjana Srdic-Rajic [3,*], Katarina Nikolic [1,*] and Slavica Oljacic [1]

[1] Department of Pharmaceutical Chemistry, Faculty of Pharmacy, University of Belgrade, Vojvode Stepe 450, 11221 Belgrade, Serbia; milan.beljkas@pharmacy.bg.ac.rs (M.B.); slavica.oljacic@pharmacy.bg.ac.rs (S.O.)
[2] Department of Organic Chemistry, Faculty of Pharmacy, University of Belgrade, Vojvode Stepe 450, 11221 Belgrade, Serbia; milos.petkovic@pharmacy.bg.ac.rs
[3] Department of Experimental Oncology, Institute for Oncology and Radiology of Serbia, Pasterova 14, 11000 Belgrade, Serbia; radovanovica@ncrc.ac.rs (A.V.); ana.djuric@ncrc.ac.rs (A.D.)
[4] Group for Molecular Oncology, Institute for Medical Research, National Institute of the Republic of Serbia, University of Belgrade, Dr. Subotica 4, 11129 Belgrade, Serbia; jfsantibanez@imi.bg.ac.rs
* Correspondence: tatjana.srdic@ncrc.ac.rs (T.S.-R.); knikolic@pharmacy.bg.ac.rs (K.N.)

S1. Supplementary Materials

S1.1. 3D-QSAR study

Table S1. Distribution of compounds in the training and test set along with the experimentally obtained and the pIC_{50} values predicted by the created 3D-QSAR (ROCK1) model

**Training set**			**Test set**		
	**pIC_{50} (exp)**	**pIC_{50} (pred)**		**pIC_{50} (exp)**	**pIC_{50} (pred)**
CHEMBL2023148	3.800	4.433	**CHEMBL2022282**	4.140	4.239
CHEMBL67352	4.120	3.956	**CHEMBL2023149**	4.670	5.260
CHEMBL2023147	4.230	4.103	**CHEMBL2023157**	5.040	6.116
CHEMBL2023161	4.510	5.098	**CHEMBL2023151**	5.540	6.286
CHEMBL225282	4.860	5.203	**CHEMBL2023153**	5.660	5.463
CHEMBL2023150	5.340	5.969	**CHEMBL2023156**	5.940	5.704
CHEMBL2023152	5.580	5.898	**CHEMBL2023154**	6.190	5.253
CHEMBL2023159	5.580	5.214	**CHEMBL4522042**	6.550	6.073
CHEMBL2023155	5.610	5.402	**CHEMBL1922044**	6.920	6.582
CHEMBL2023158	5.770	5.664	**CHEMBL1922033**	7.340	7.192
CHEMBL2023160	5.850	5.753	**CHEMBL1084106**	7.410	6.879
CHEMBL1922030	5.960	5.897	**CHEMBL1922133**	7.520	7.533
CHEMBL1922028	6.150	5.711	**CHEMBL1922026**	7.720	7.055
CHEMBL38380	6.180	6.192	**CHEMBL1922132**	7.960	8.050
CHEMBL4555093	6.510	6.495	**CHEMBL1922129**	8.30	7.537
CHEMBL1084890	6.750	6.358			
CHEMBL1922143	7.040	7.443			
CHEMBL1922134	7.120	6.714			
CHEMBL1085141	7.230	7.565			
CHEMBL1084900	7.280	7.981			
CHEMBL1922142	7.410	7.581			
CHEMBL1922043	7.480	7.653			
CHEMBL1922042	7.570	7.986			
CHEMBL1922125	7.680	8.133			
CHEMBL1084107	7.720	6.732			
GSK	7.850	7.587			
CHEMBL1084892	7.850	7.744			
CHEMBL1922126	7.890	7.397			
CHEMBL1922034	7.920	7.397			
CHEMBL1922127	8.050	7.914			
RKI1447	8.100	7.603			
CHEMBL1922140	8.220	7.956			
CHEMBL1922128	8.520	8.678			
CHEMBL1922035	8.700	8.637			

Table S2. Distribution of compounds in the training and test set along with the experimentally obtained and the pIC_{50} values predicted by the created 3D-QSAR (ROCK2) model

**Training set**			**Test set**		
	**pIC_{50} (exp)**	**pIC_{50} (pred)**		**pIC_{50}(exp)**	**pIC_{50} (pred)**
CHEMBL2023148	4.210	4.353	**CHEMBL2023147**	4.440	4.908
CHEMBL2023161	4.480	5.122	**CHEMBL3689476**	4.830	5.336
CHEMBL2023159	4.700	4.830	**CHEMBL2023157**	5.120	5.247
CHEMBL2023149	5.120	5.322	**CHEMBL3689479**	5.380	5.008
CHEMBL225282	5.260	5.018	**CHEMBL3689466**	5.590	6.052
CHEMBL2023160	5.270	5.093	**CHEMBL2023153**	5.960	5.941
CHEMBL3689496	5.270	5.254	**CHEMBL3689482**	6.200	5.895
CHEMBL3689498	5.400	5.533	**CHEMBL2023156**	6.580	6.315
CHEMBL2023150	5.640	6.324	**CHEMBL3689502**	6.950	6.757
CHEMBL2023151	5.820	5.985	**CHEMBL1922135**	7.210	6.617
CHEMBL3689477	5.830	5.693	**CHEMBL2006299**	7.680	7.41
CHEMBL2023155	5.970	5.816	**CHEMBL1922044**	7.750	7.47
CHEMBL3689484	6.090	6.310	**CHEMBL1922033**	8.000	7.597
CHEMBL2023152	6.100	6.078	**CHEMBL1922133**	8.220	8.017
CHEMBL2023154	6.170	5.724	**CHEMBL1922132**	8.700	7.289
CHEMBL38380	6.400	5.965			
CHEMBL3689506	6.420	6.535			
CHEMBL3689504	6.560	6.435			
CHEMBL1922030	6.700	6.478			
CHEMBL3689500	6.800	7.208			
CHEMBL2023158	7.000	6.533			
CHEMBL3689470	7.130	7.254			
GSK	7.200	7.020			
CHEMBL4522042	7.430	7.257			
CHEMBL1922134	7.720	7.315			
CHEMBL1922143	7.800	8.686			
CHEMBL1922043	8.100	8.113			
RKI1447	8.210	7.762			
CHEMBL1922125	8.300	7.929			
CHEMBL1922042	8.400	8.325			
CHEMBL1922034	8.700	8.781			
CHEMBL1922128	9.000	9.086			

Table S3. Structures of ROCK1 and ROCK2 inhibitors that are used for 3D-QSAR and molecular docking studies

**Compound**	**Structure**
CHEMBL1084106	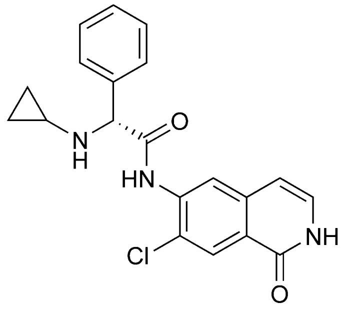
CHEMBL1084107	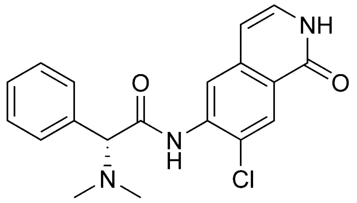
CHEMBL1084890	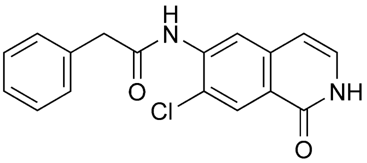
CHEMBL1084892	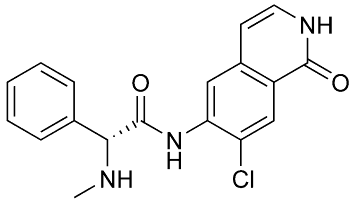
CHEMBL1084900	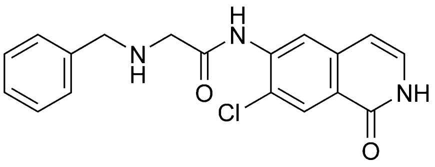
CHEMBL1085141	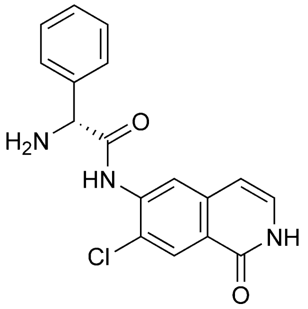

**Compound**	**Structure**
CHEMBL1922026	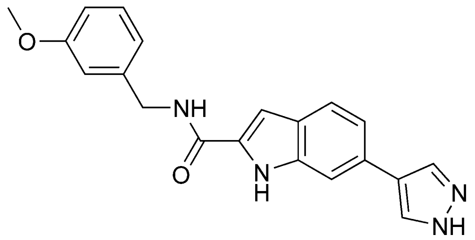
CHEMBL1922028	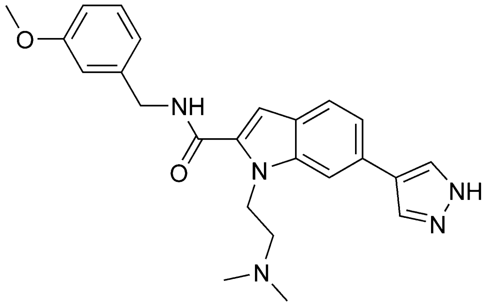
CHEMBL1922030	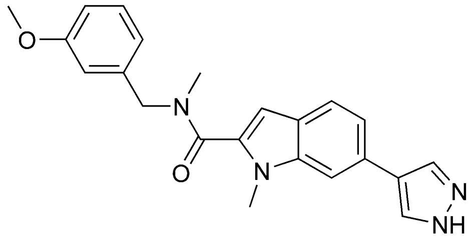
CHEMBL1922033	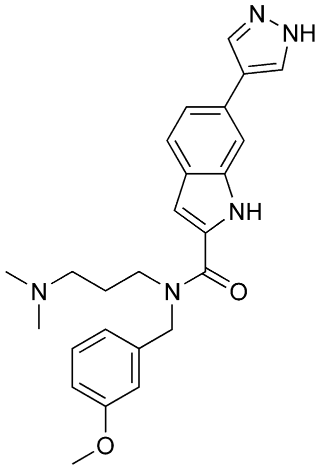
CHEMBL1922034	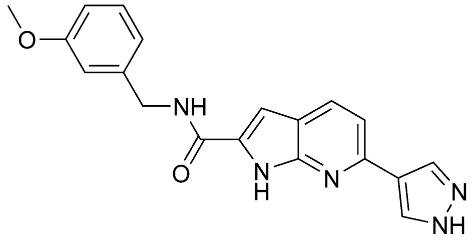

**Compound**	**Structure**
CHEMBL1922035	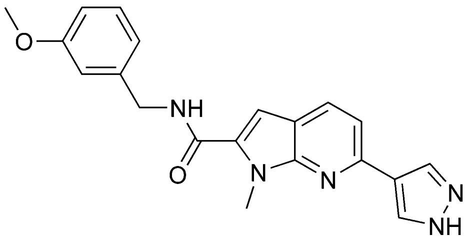
CHEMBL1922042	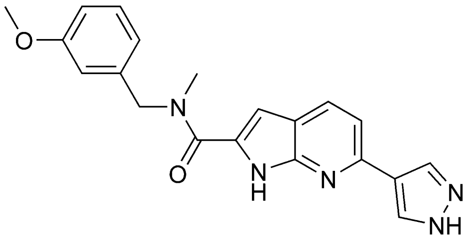
CHEMBL1922043	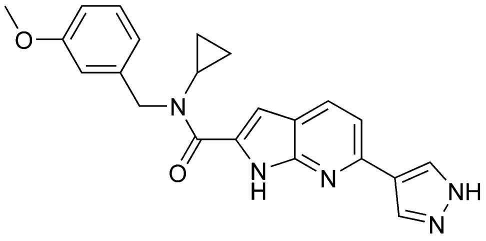
CHEMBL1922044	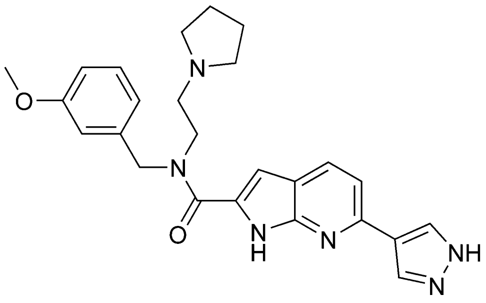
CHEMBL1922125	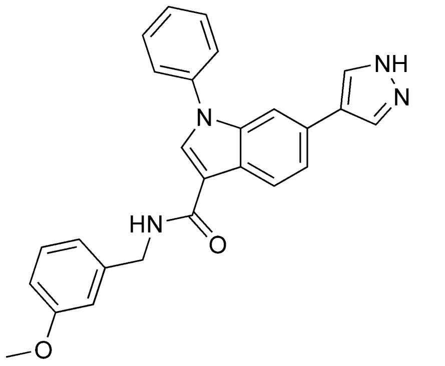

Compound	Structure
CHEMBL1922126	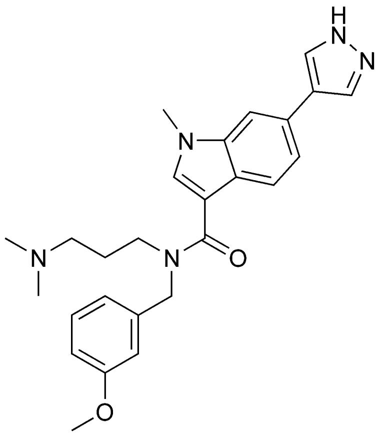
CHEMBL1922127	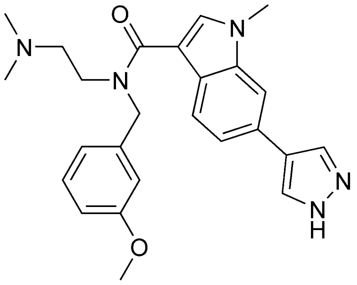
CHEMBL1922128	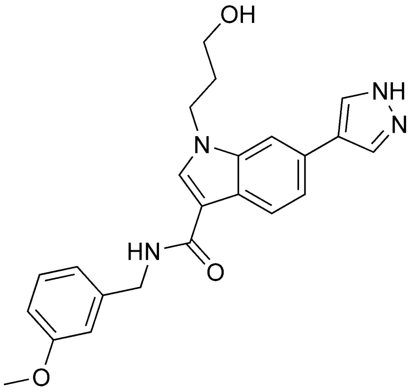
CHEMBL1922129	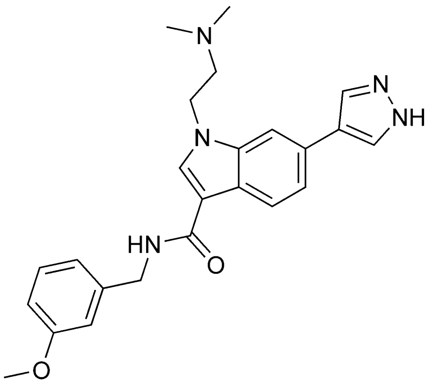

**Compound**	**Structure**
CHEMBL1922132	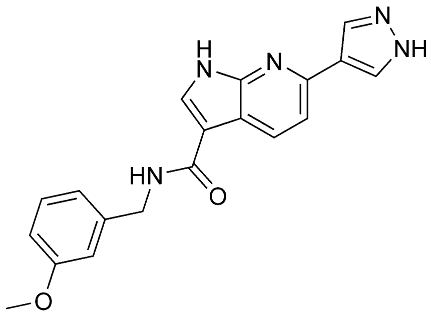
CHEMBL1922133	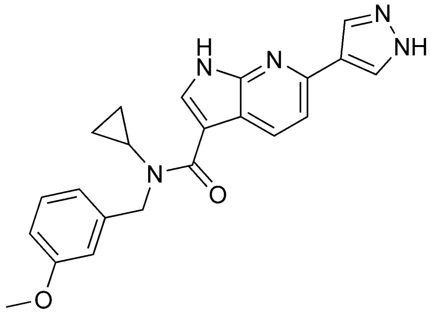
CHEMBL1922134	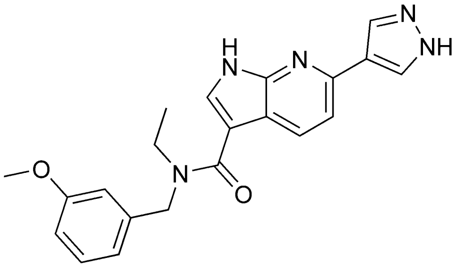
CHEMBL1922135	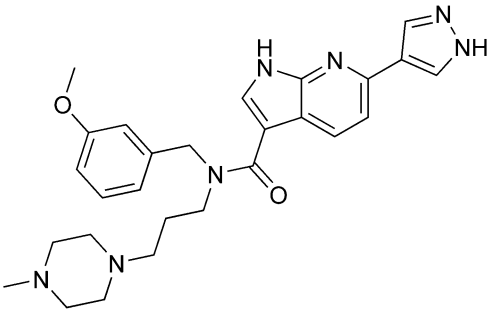
CHEMBL1922140	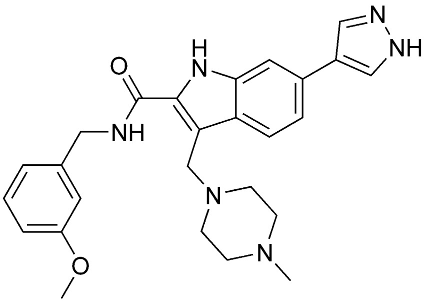

Compound	Structure
CHEMBL1922142	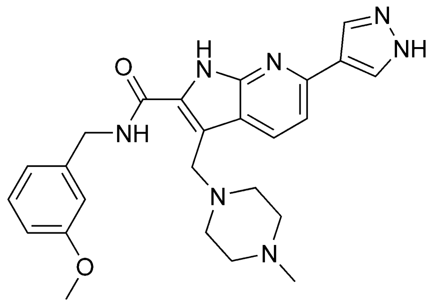
CHEMBL1922143	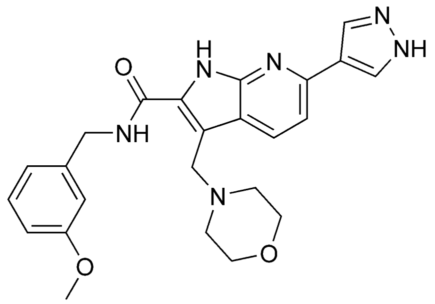
CHEMBL2006299	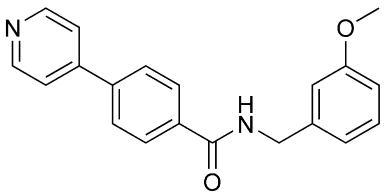
CHEMBL2022282	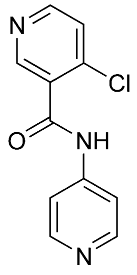
CHEMBL2023147	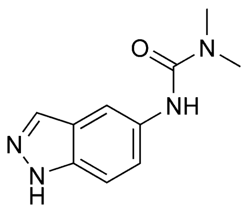

**Compound**	**Structure**
CHEMBL2023148	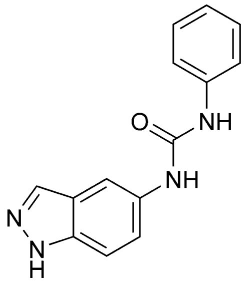
CHEMBL2023149	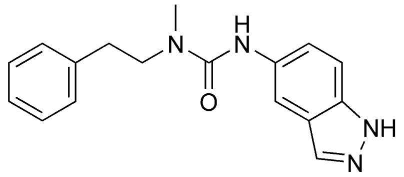
CHEMBL2023150	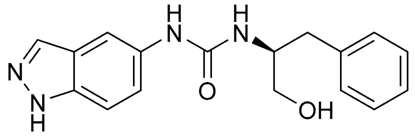
CHEMBL2023151	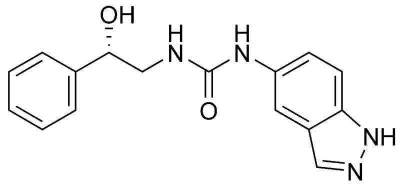
CHEMBL2023152	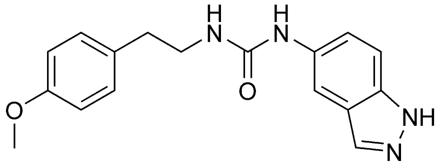
CHEMBL2023153	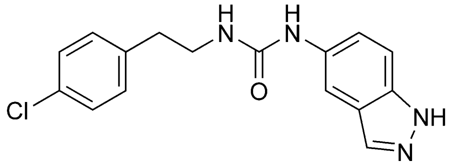
CHEMBL2023154	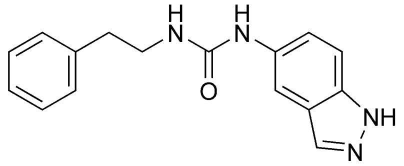
CHEMBL2023155	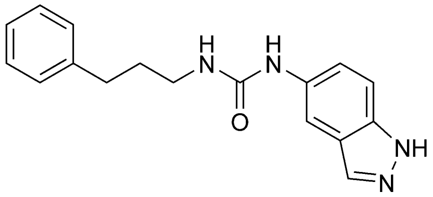

**Compound**	**Structure**
CHEMBL2023156	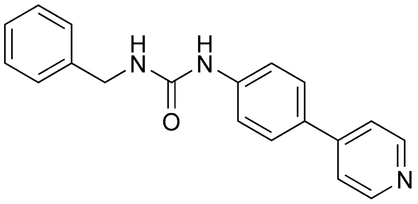
CHEMBL2023157	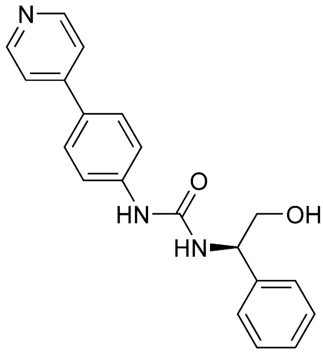
CHEMBL2023158	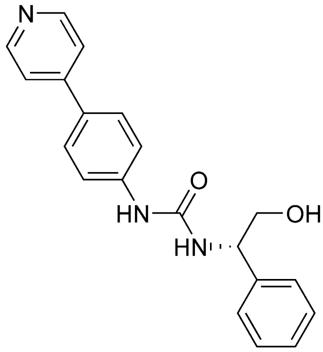
CHEMBL2023159	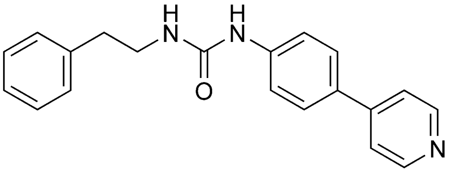
CHEMBL2023160	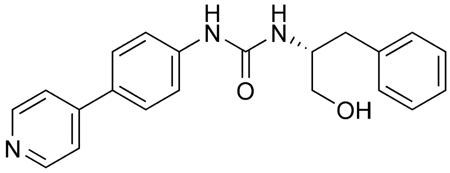
CHEMBL2023161	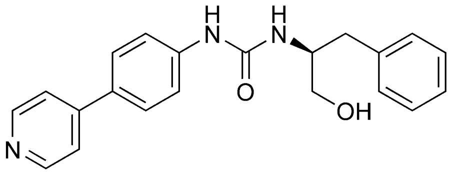

**Compound**	**Structure**
CHEMBL225282	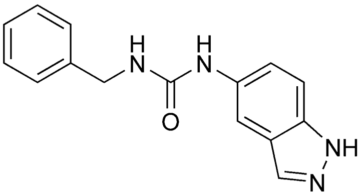
CHEMBL3689466	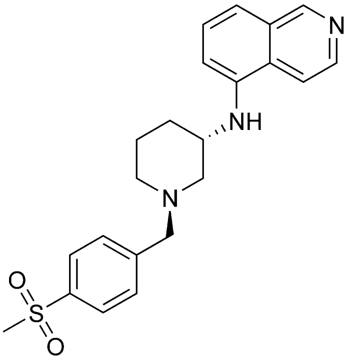
CHEMBL3689470	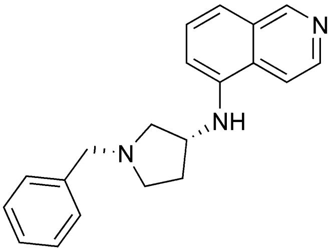
CHEMBL3689476	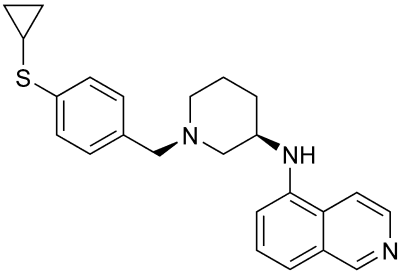
CHEMBL3689477	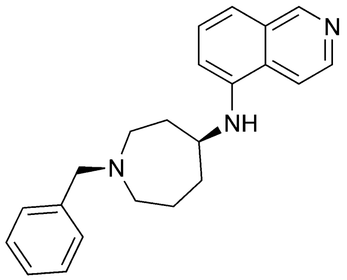

Compound	Structure
CHEMBL3689479	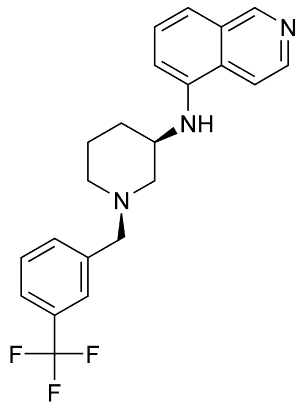
CHEMBL3689482	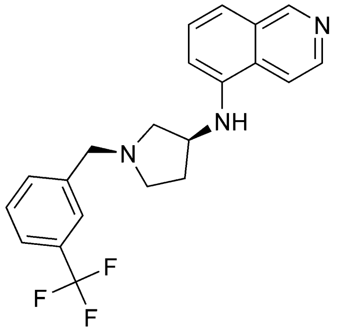
CHEMBL3689484	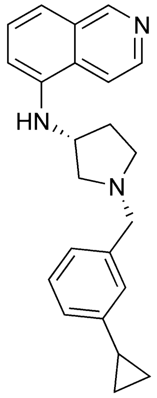
CHEMBL3689496	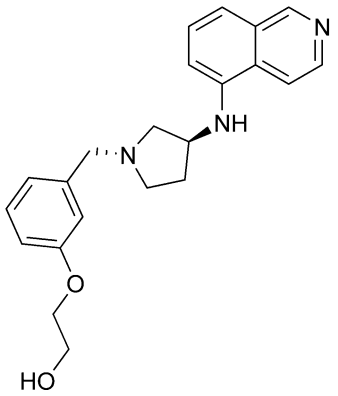

Compound	Structure
CHEMBL3689498	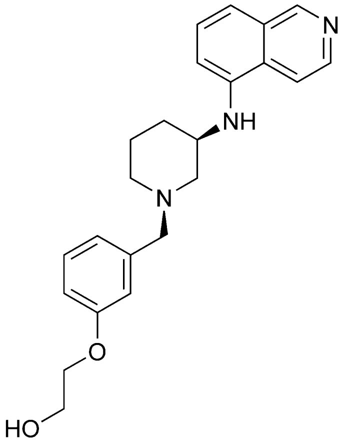
CHEMBL3689500	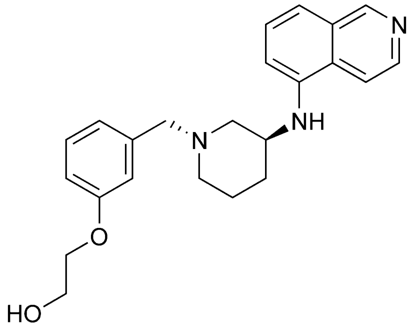
CHEMBL3689502	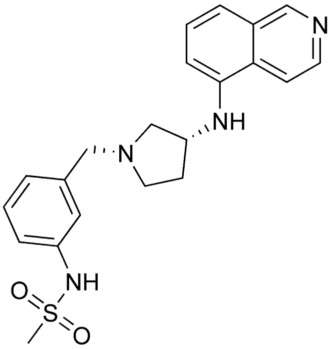
CHEMBL3689504	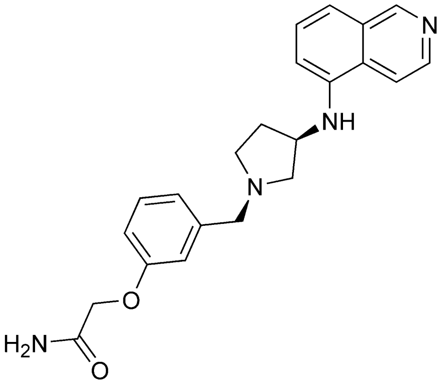

Compound	Structure
CHEMBL3689506	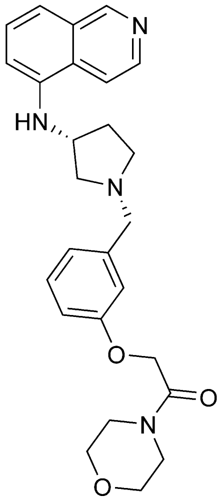
CHEMBL38380	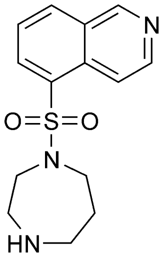
CHEMBL4522042	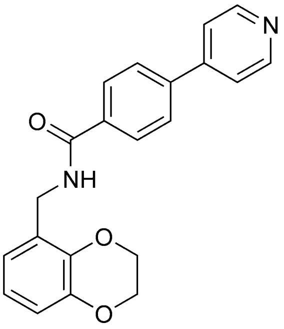
CHEMBL4555093	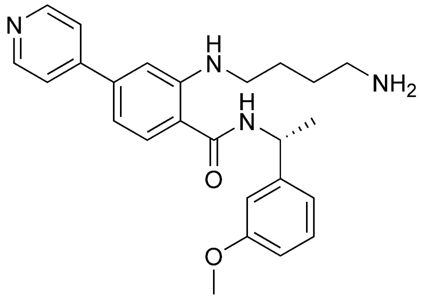

**Compound**	**Structure**
CHEMBL67352	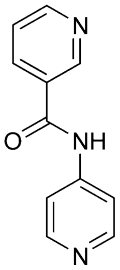
GSK	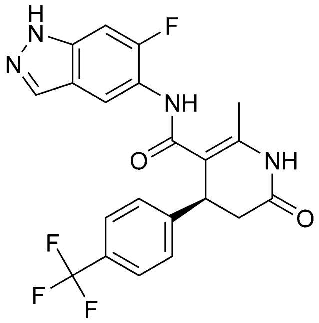
RKI1447	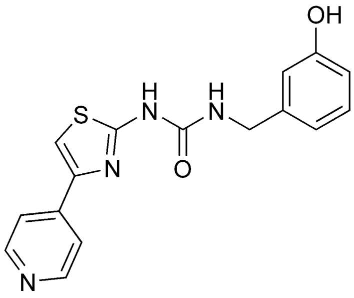

Table S4. Predicted activity of designed comounds

**Compound**	**pIC_{50} (ROCK1-predicted)**	**pIC_{50} (ROCK2-predicted)**
C-19	5.76	5.61
C-20	7.21	6.74
C-21	6.80	6.17
C-22	5.99	7.44
C-23	7.05	7.12
C-24	5.88	6.25
C-25	7.30	7.87
C-26	7.18	6.22
C-27	5.25	6.35

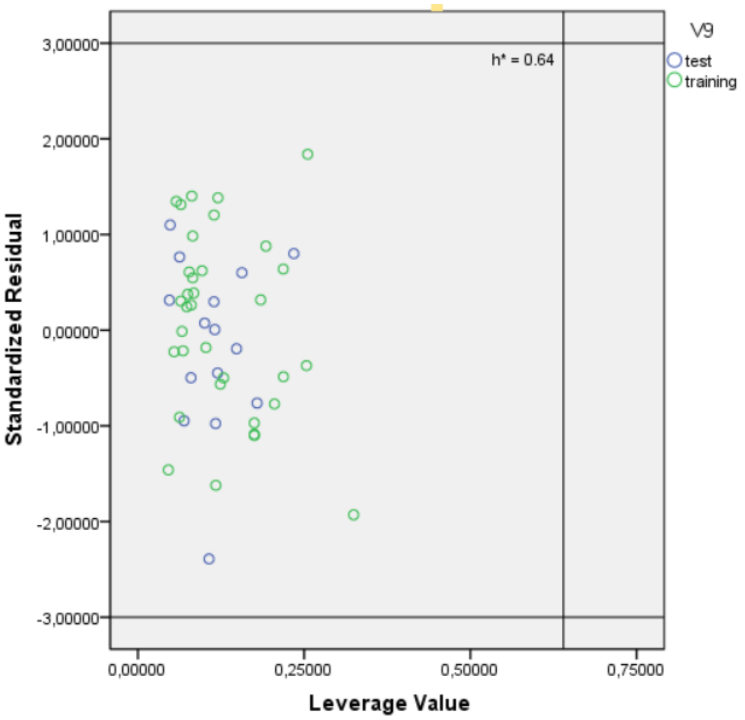

Applicability domain of 3D-QSAR(ROCK1) model

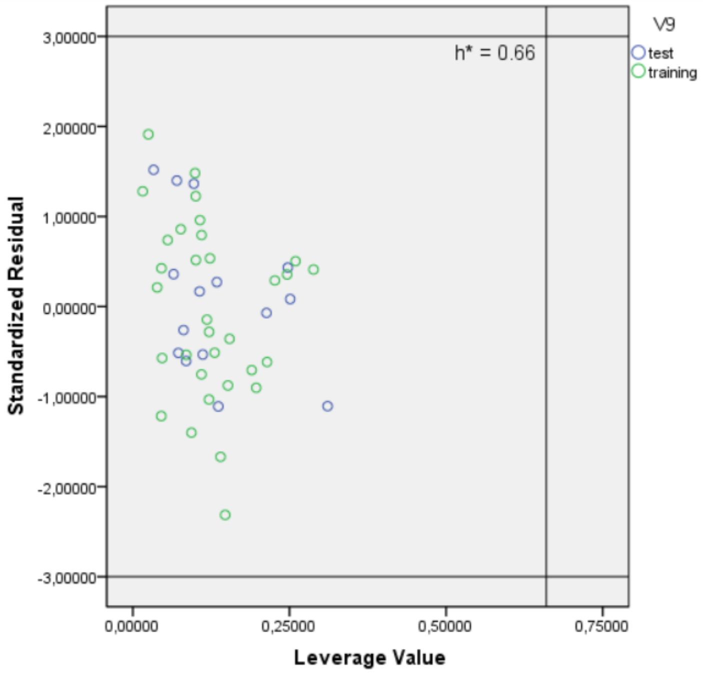

Applicability domain of 3D-QSAR(ROCK2) model

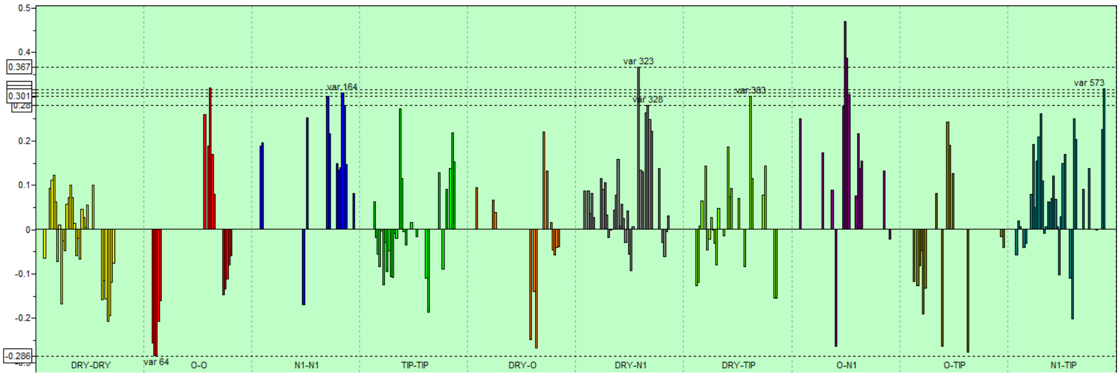

PLS coefficient plot for the 3D-QSAR (ROCK1) model. The most significant variables are labeled.

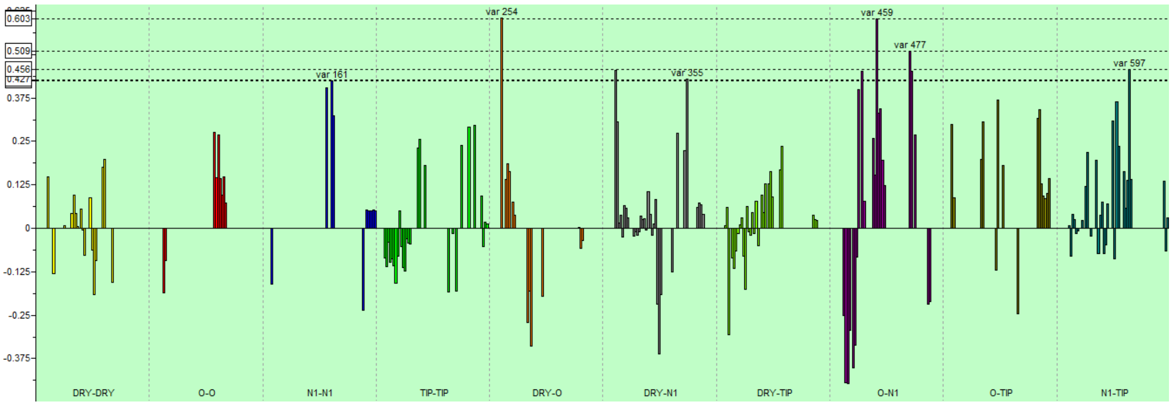

PLS coefficient plot for the 3D-QSAR (ROCK2) model. The most significant variables are labelled.

S1.2. Molecular docking study

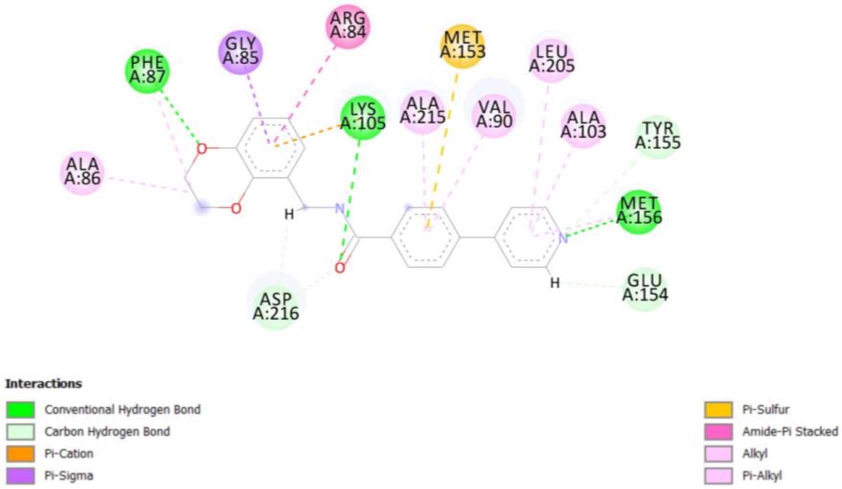

Figure S1. The diagram of the main interactions between co-crystal ligand J0P501 and ROCK1 (PDB:6E9W)

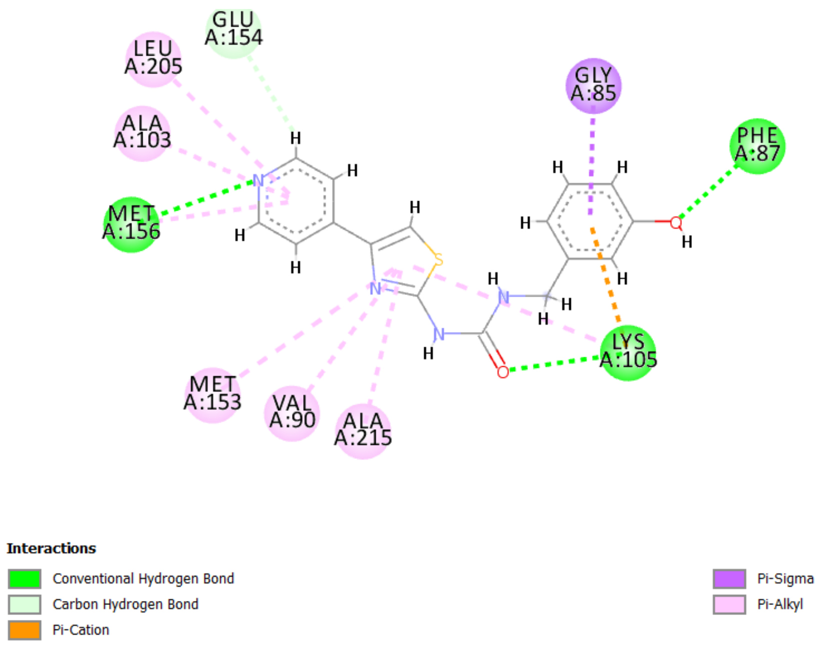

Figure S2. The diagram of the main interactions RKI1447-ROCK1 (PDB:6E9W)

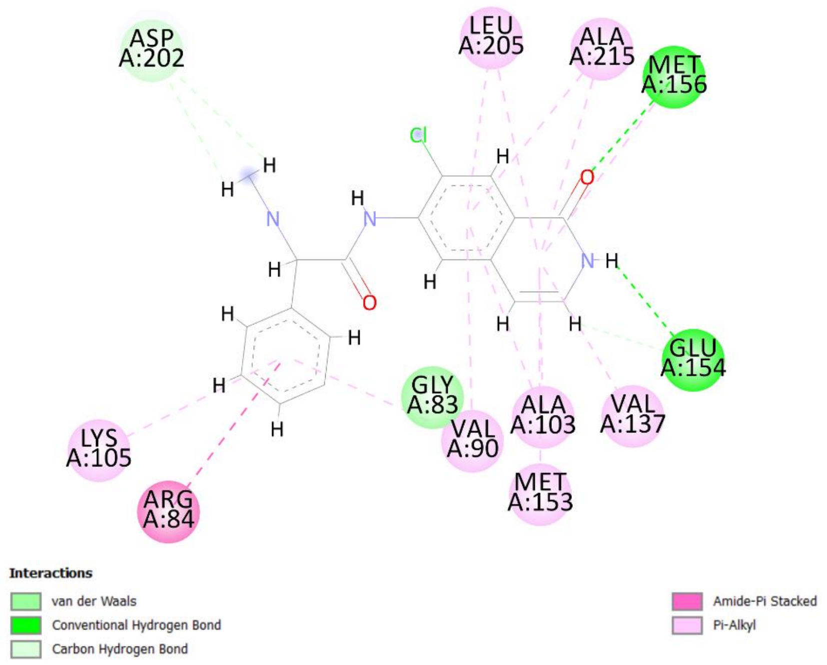

Figure S3. The diagram of the main interactions ChEMBL1084892-ROCK1 (PDB:6E9W)

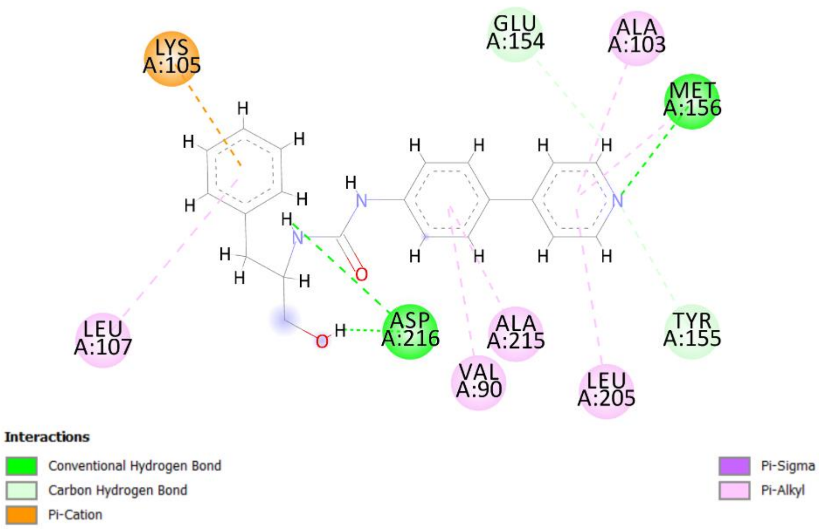

Figure S4. The diagram of the main interactions ChEMBL2023160-ROCK1 (PDB:6E9W)

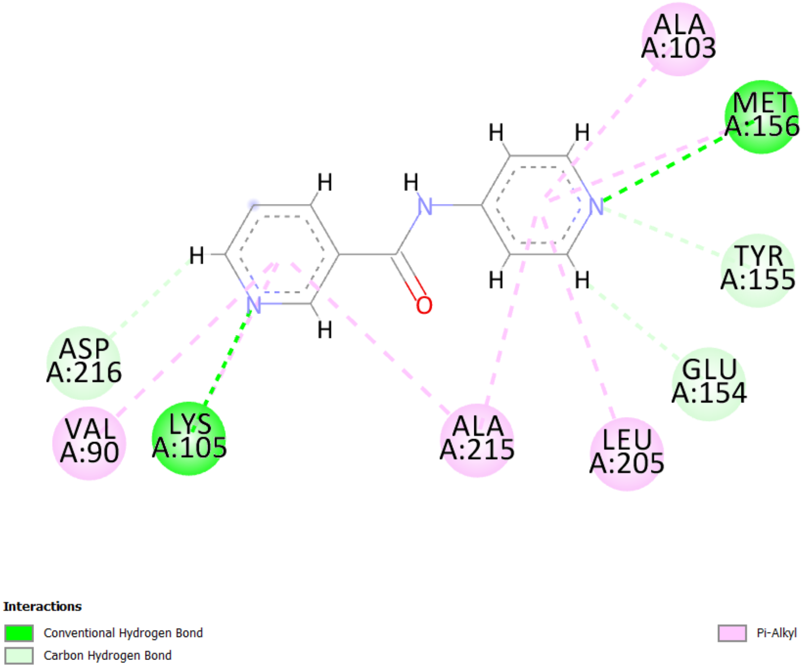

Figure S5. The diagram of the main interactions ChEMBL67352-ROCK1 (PDB:6E9W)

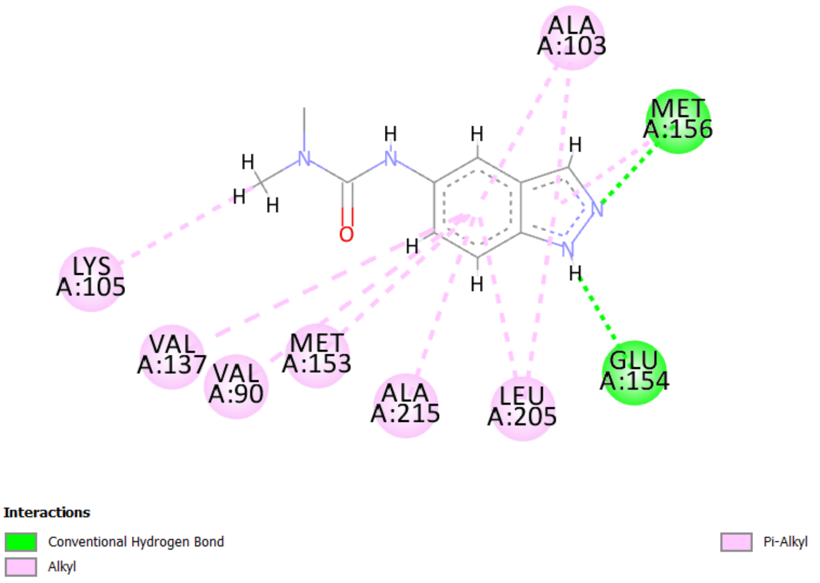

Figure S6. The diagram of the main interactions ChEMBL2023147-ROCK1 (PDB:6E9W)

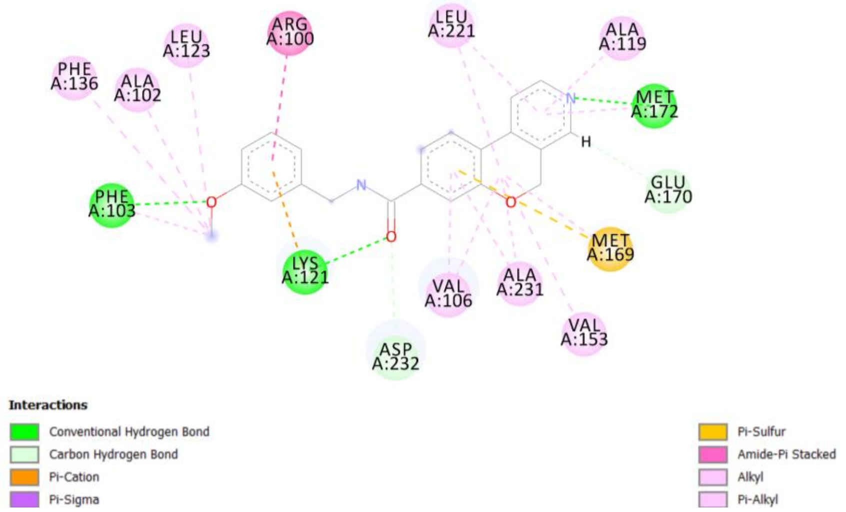

Figure S7. The diagram of the main interactions between co-crystal ligand VFA501 and ROCK2 (PDB:7JNT)

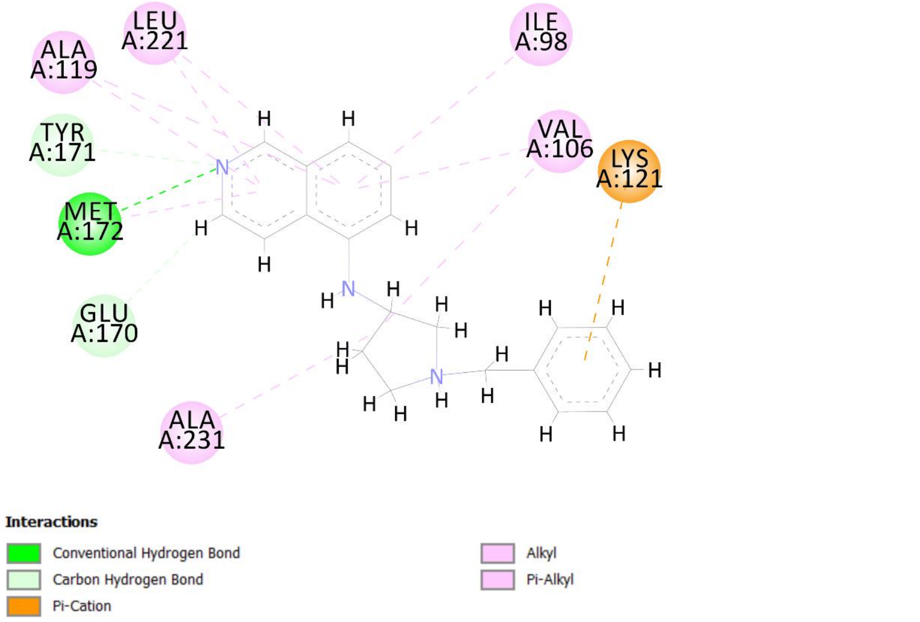

Figure S8. The diagram of the main interactions ChEMBL3689470-ROCK2 (PDB:7JNT)

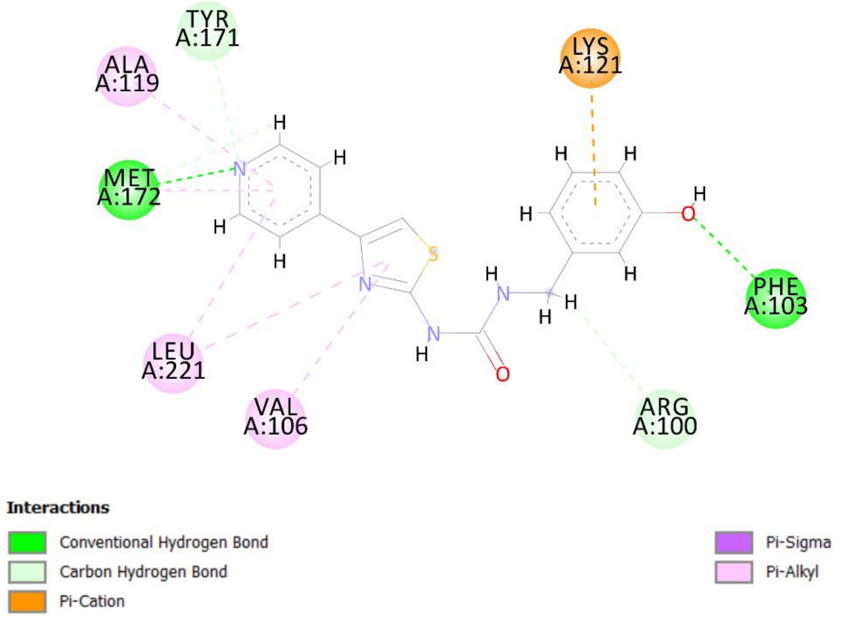

Figure S9. The diagram of the main interactions RKI1447-ROCK2 (PDB:7JNT)

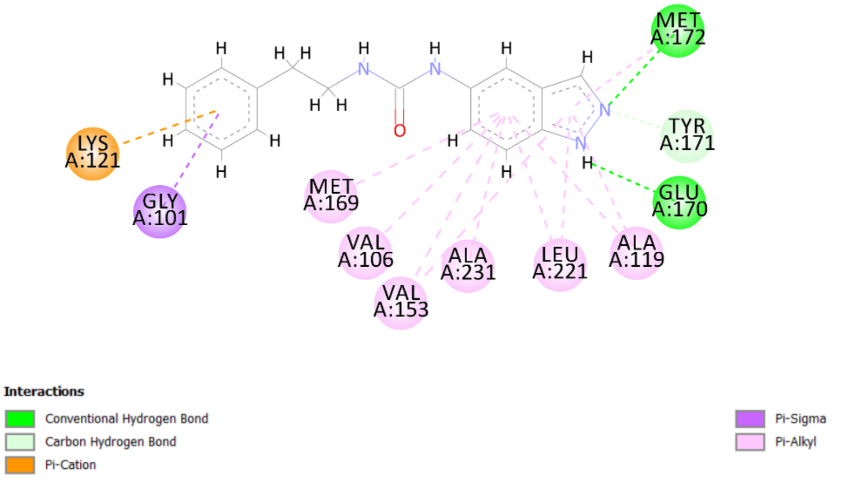

Figure S10. The diagram of the main interactions ChEMBL2023154-ROCK2 (PDB:7JNT)

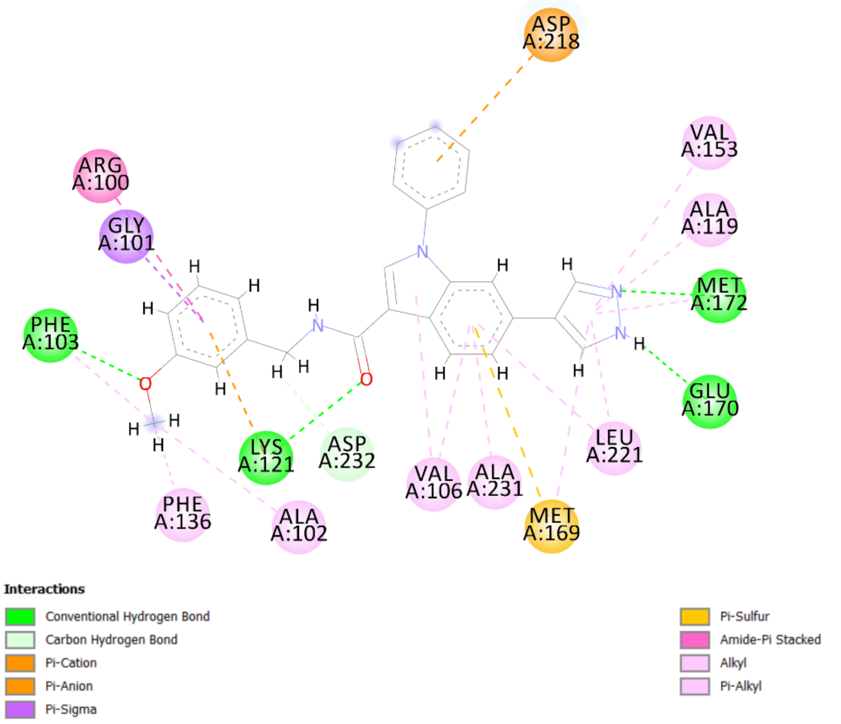

Figure S11. The diagram of the main interactions ChEMBL1922125-ROCK2 (PDB:7JNT)

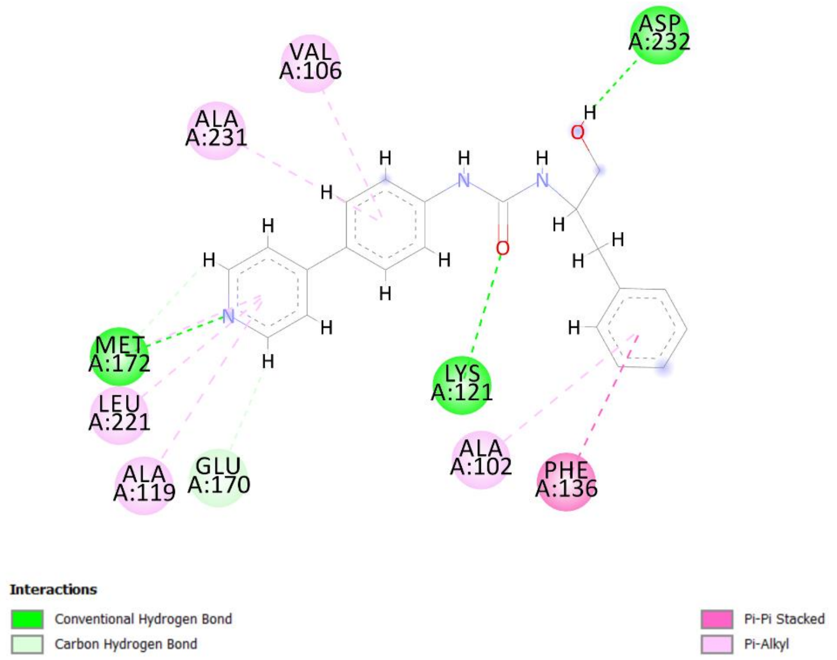

Figure S12. The diagram of the main interactions ChEMBL2023161-ROCK2 (PDB:7JNT)

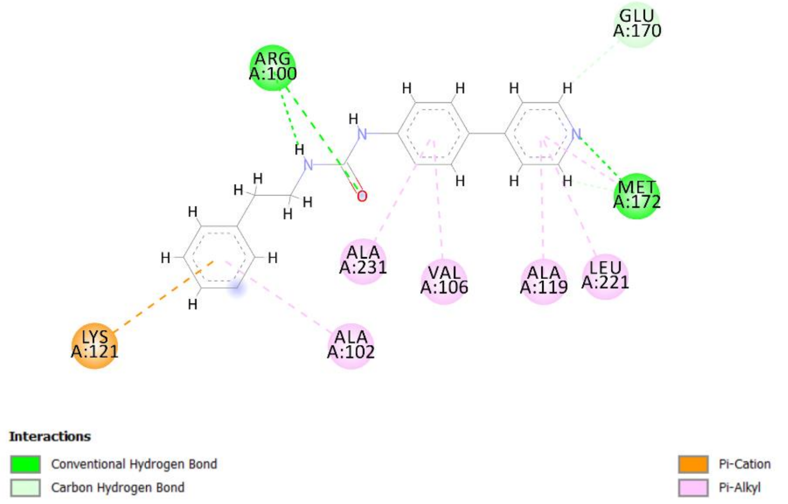

Figure S13. The diagram of the main interactions ChEMBL2023159-ROCK2 (PDB:7JNT)

S1.3. ¹H and ¹³C NMR spectra of the final compounds

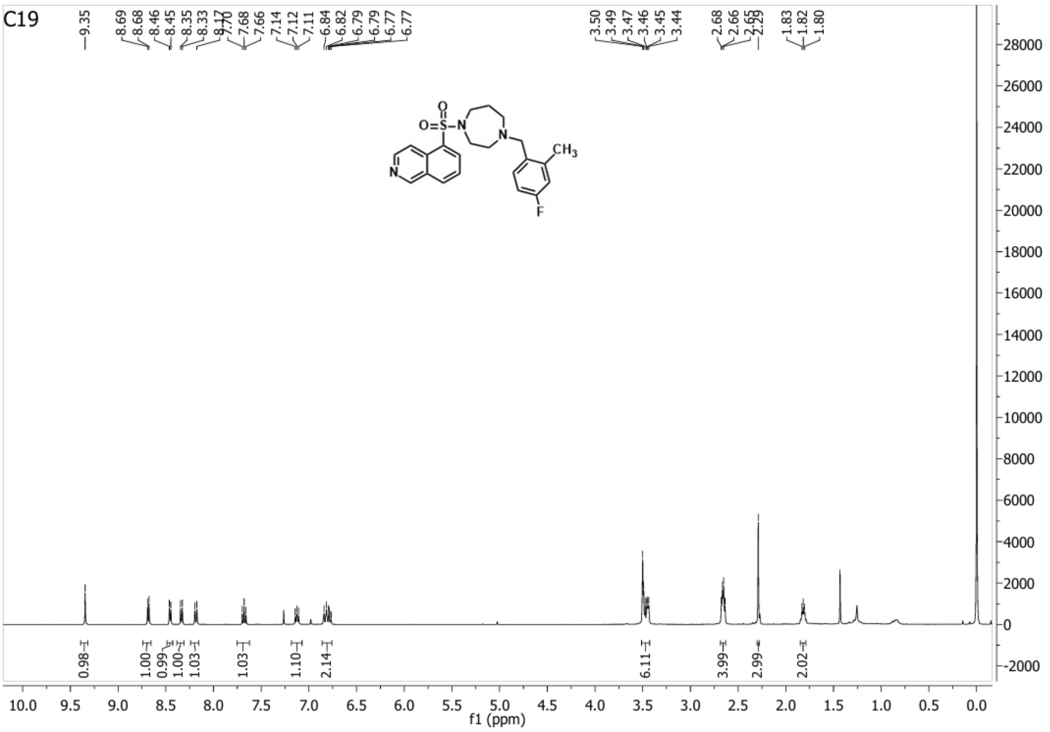

Figure S14. ¹H NMR Spectra of C-19

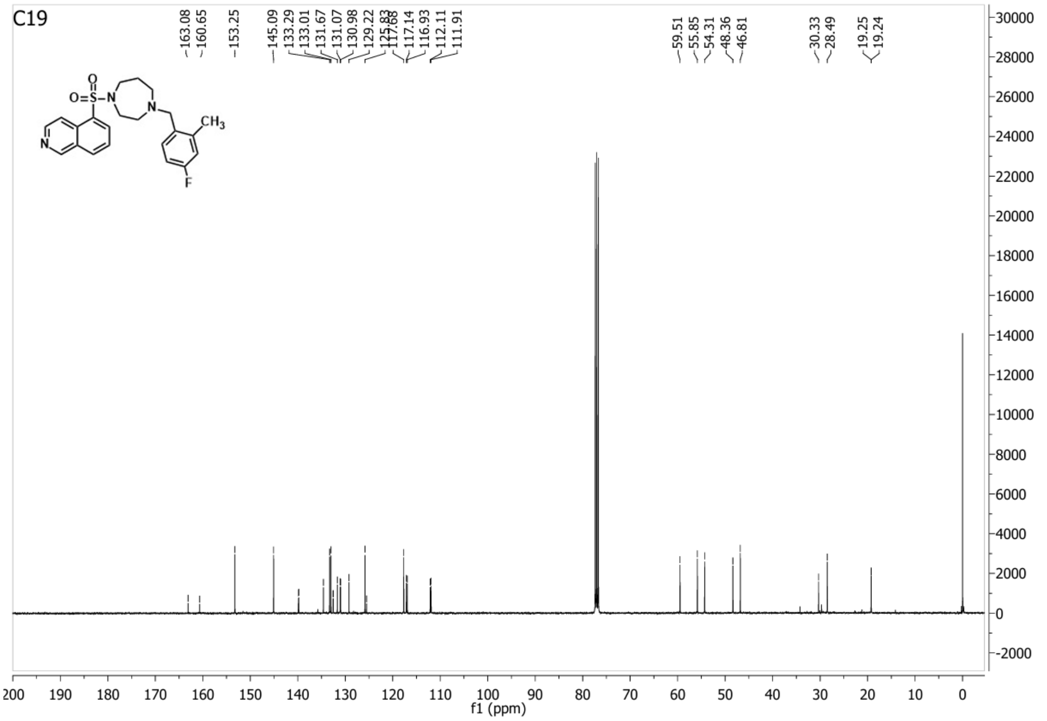

Figure S15. ¹³C NMR Spectra of C-19

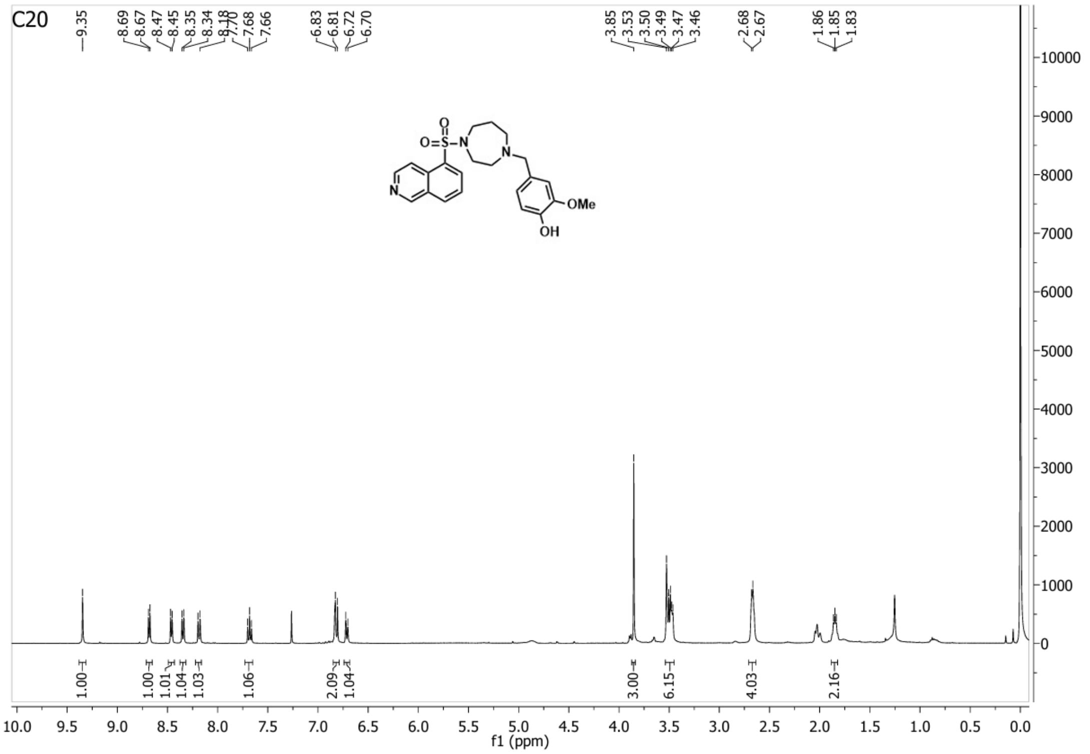

Figure S16. ^{1}H NMR Spectra of C-20

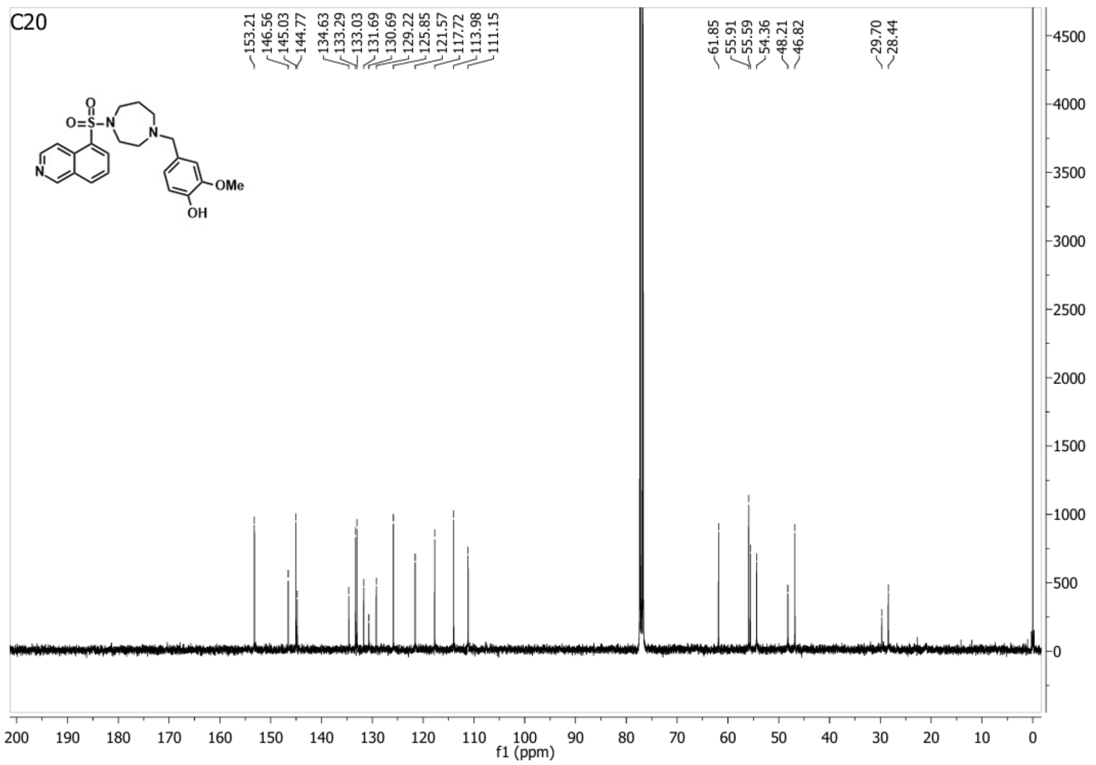

Figure S17. ^{13}C NMR Spectra of C-20

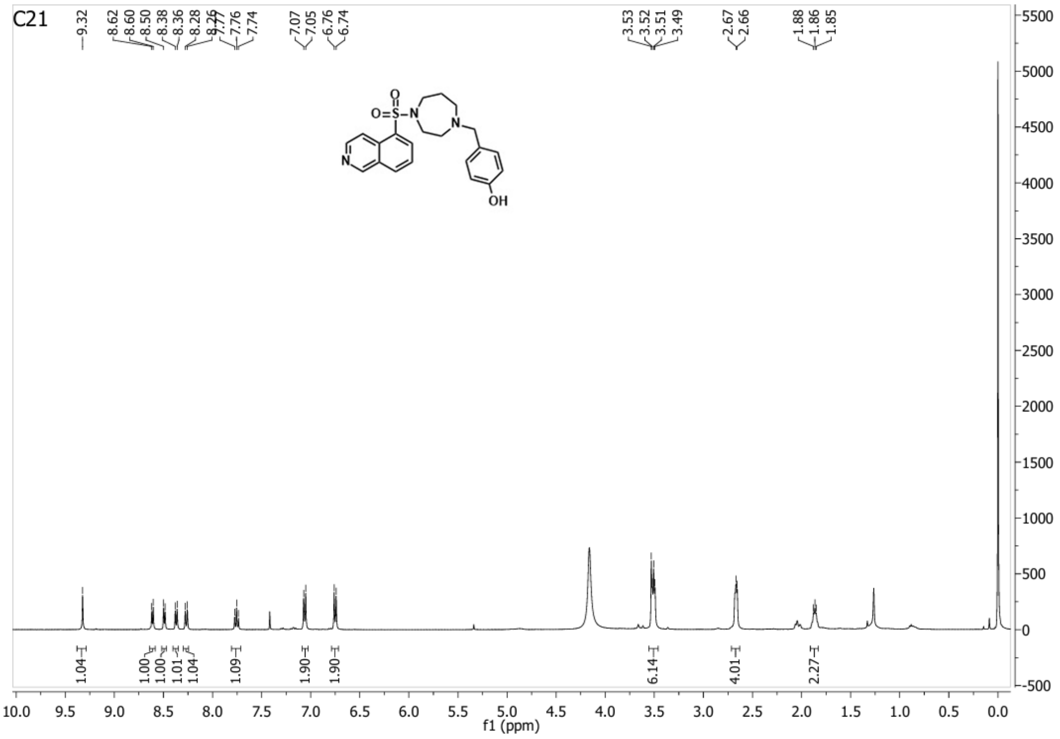

Figure S18. ^{1}H NMR Spectra of C-21

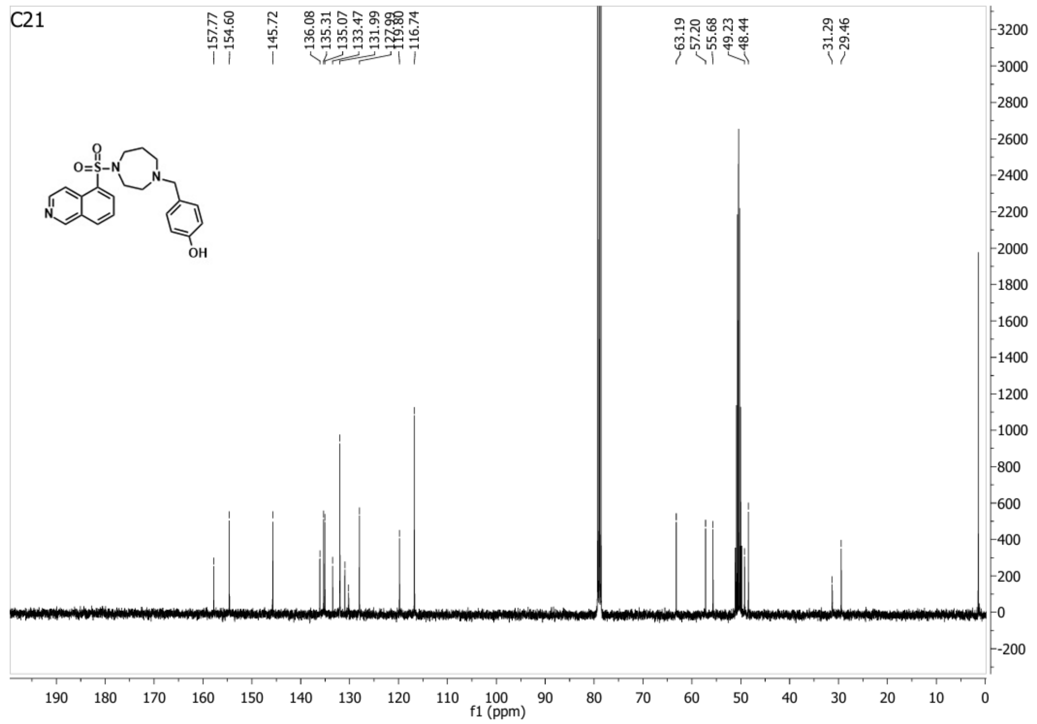

Figure S19. ^{13}C NMR Spectra of C-21

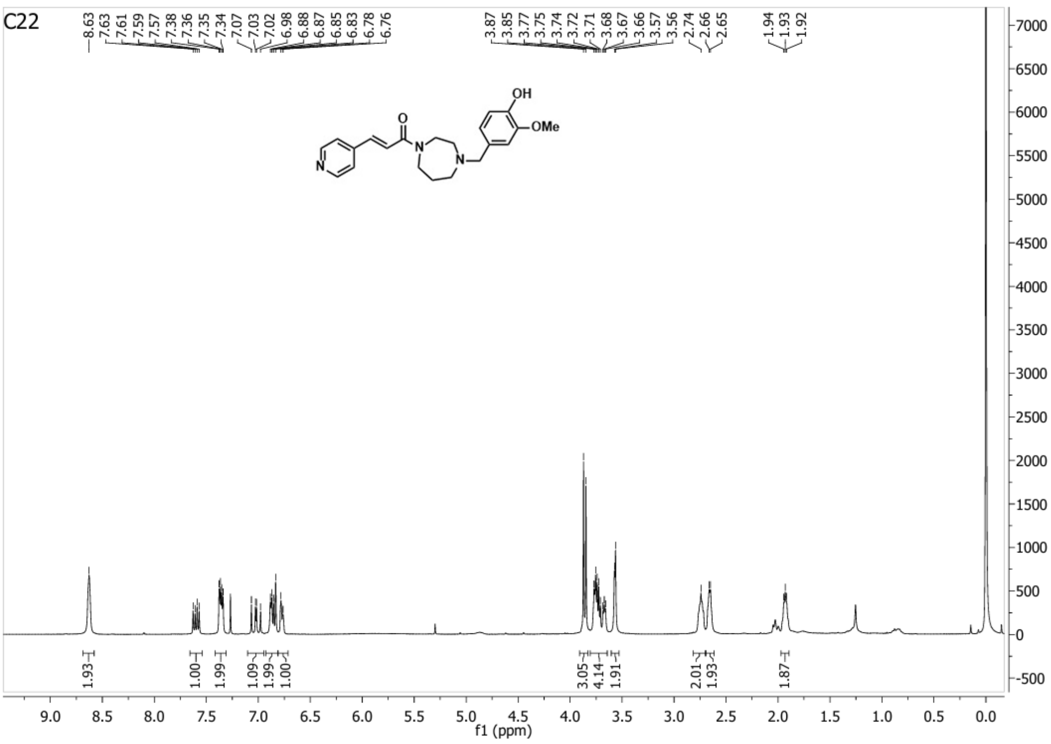

Figure S20. ^{1}H NMR Spectra of C-22

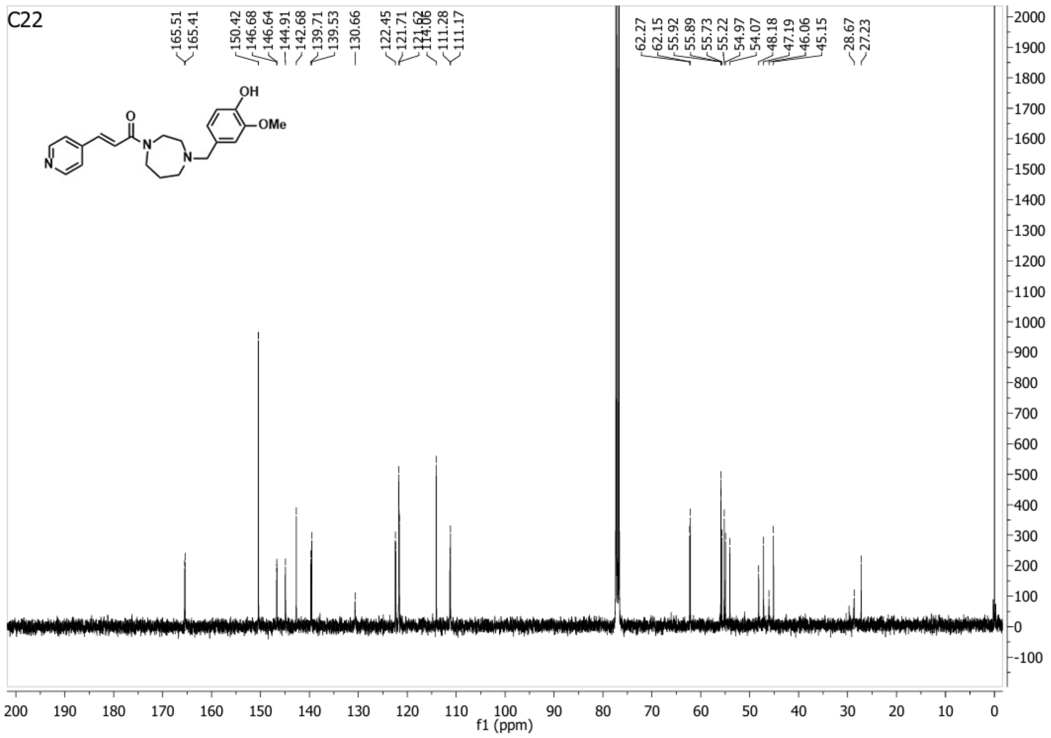

Figure S21. ^{13}C NMR Spectra of C-22

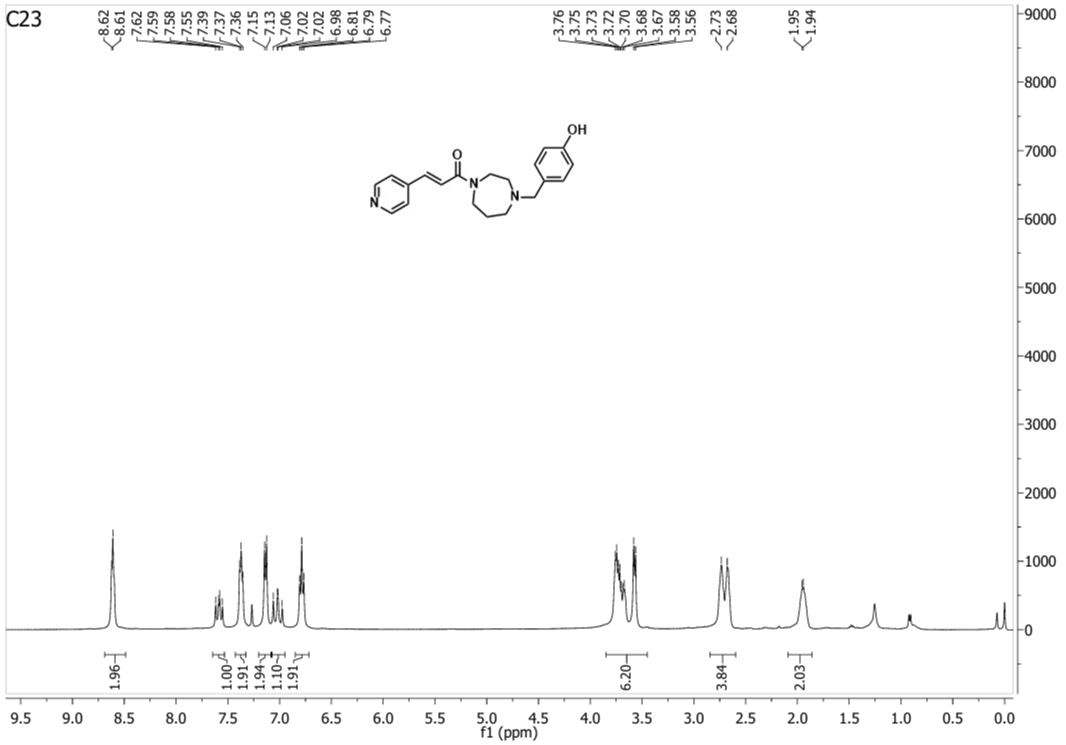

Figure S22. ^{1}H NMR Spectra of C-23

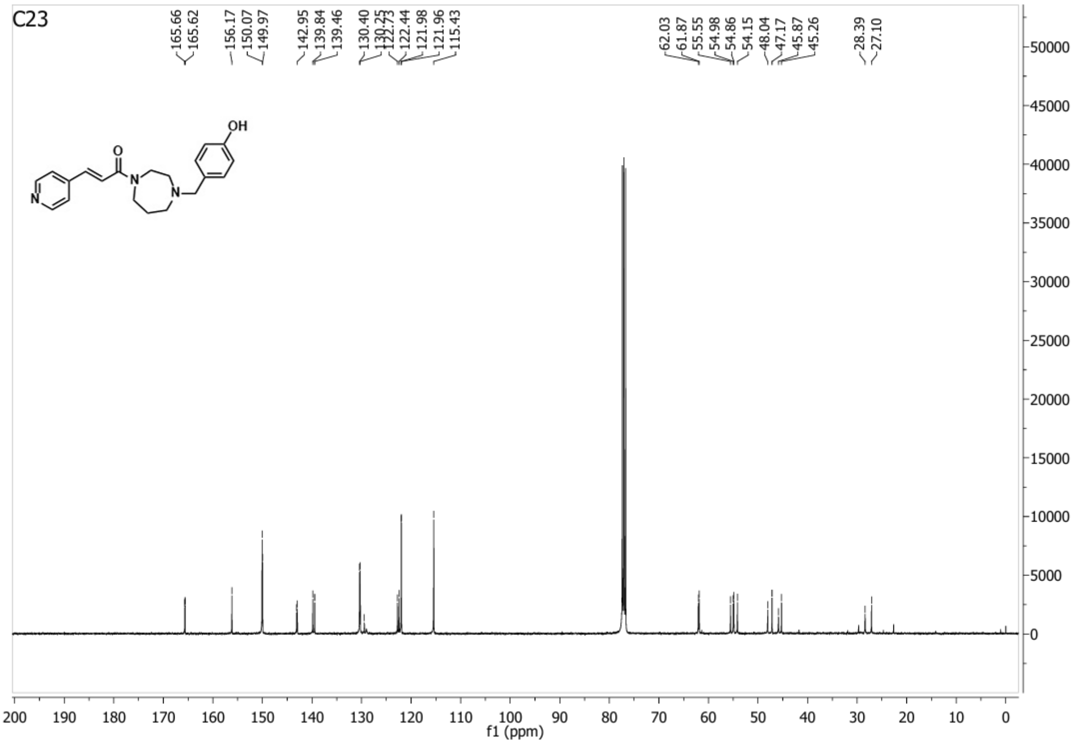

Figure S23. ^{13}C NMR Spectra of C-23

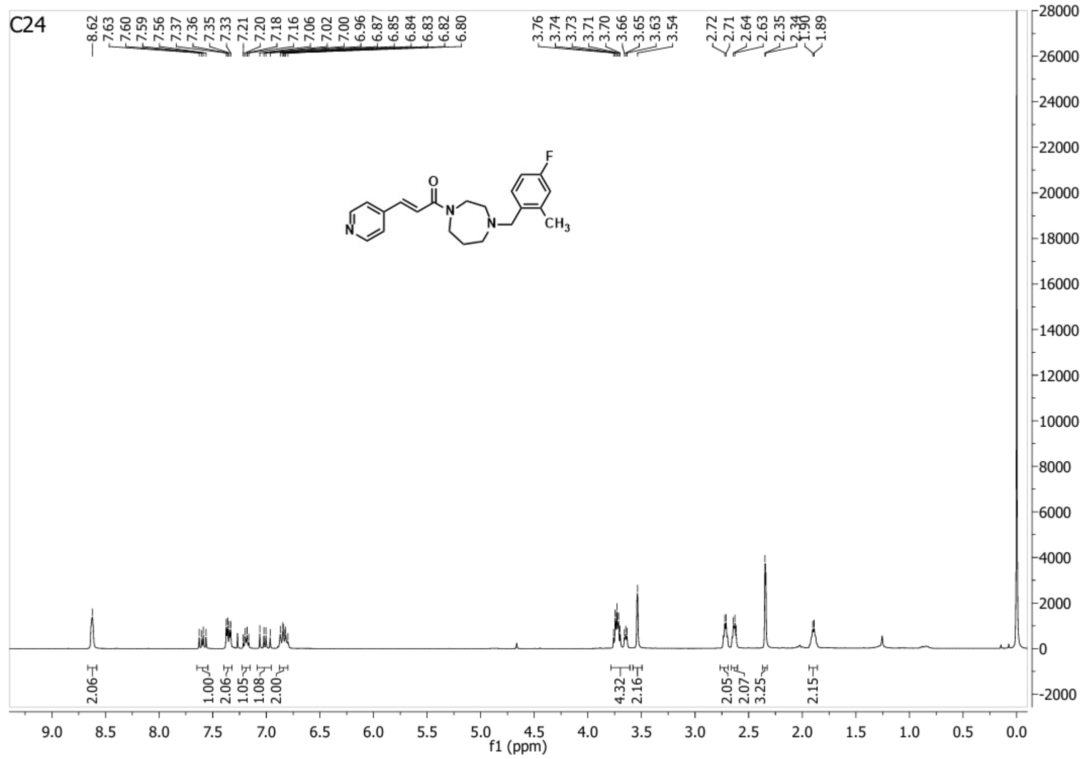

Figure S24. ^{1}H NMR Spectra of C-24

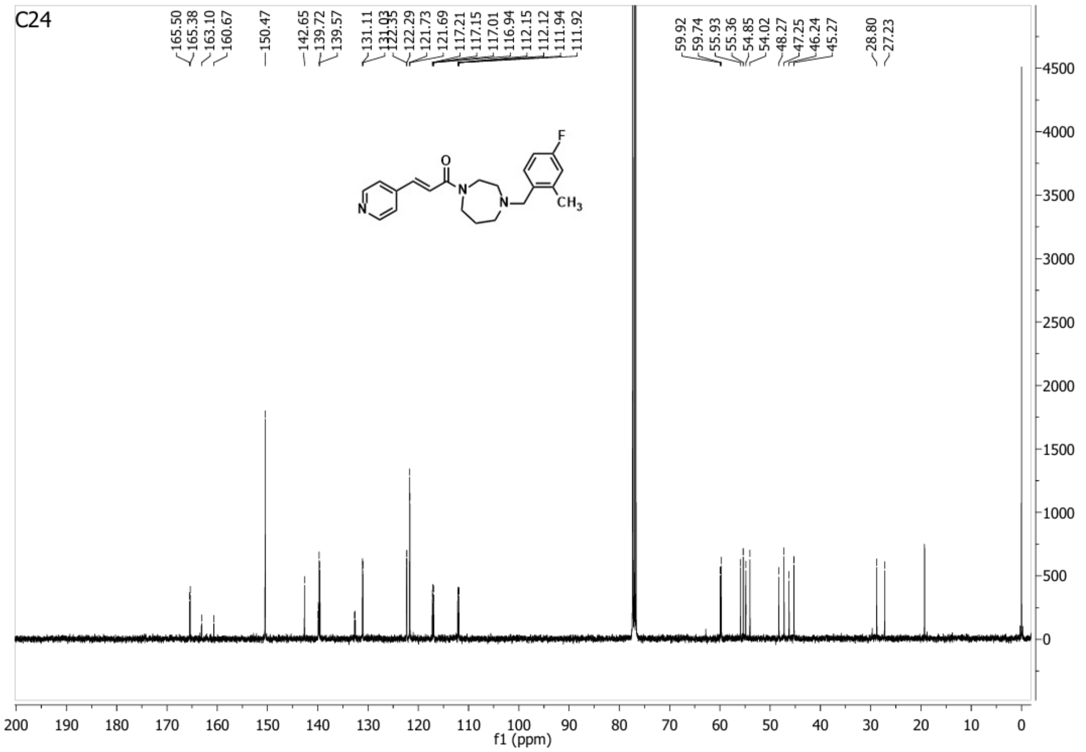

Figure S25. ^{13}C NMR Spectra of C-24

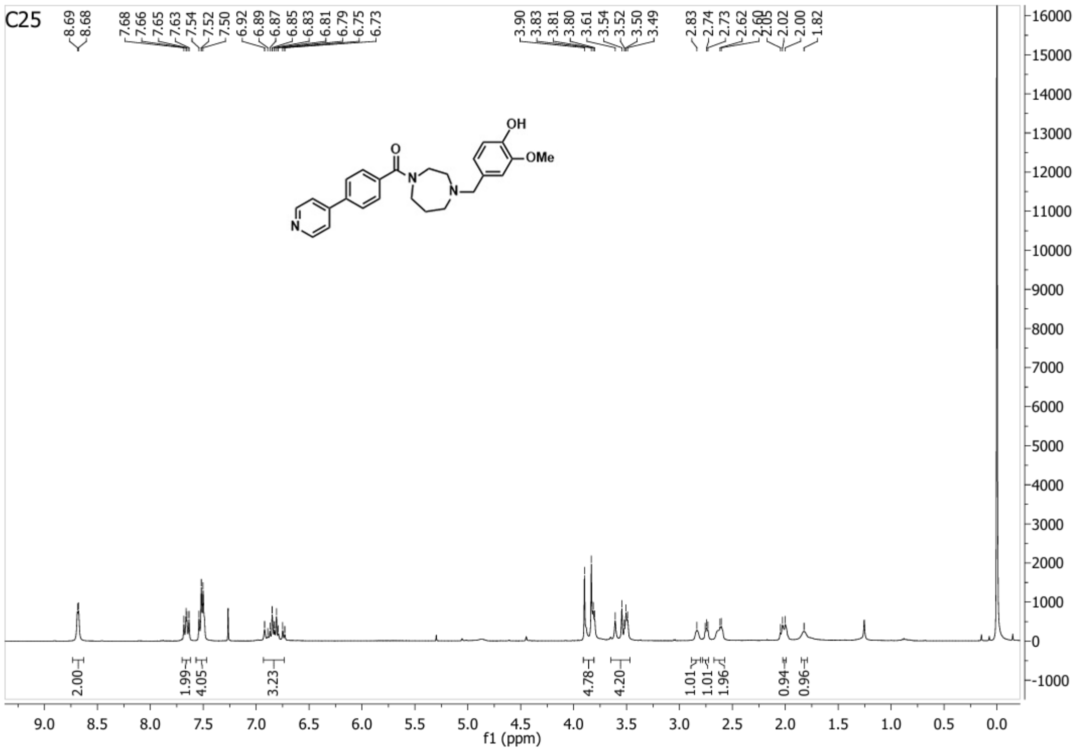

Figure S26. ^{1}H NMR Spectra of C-25

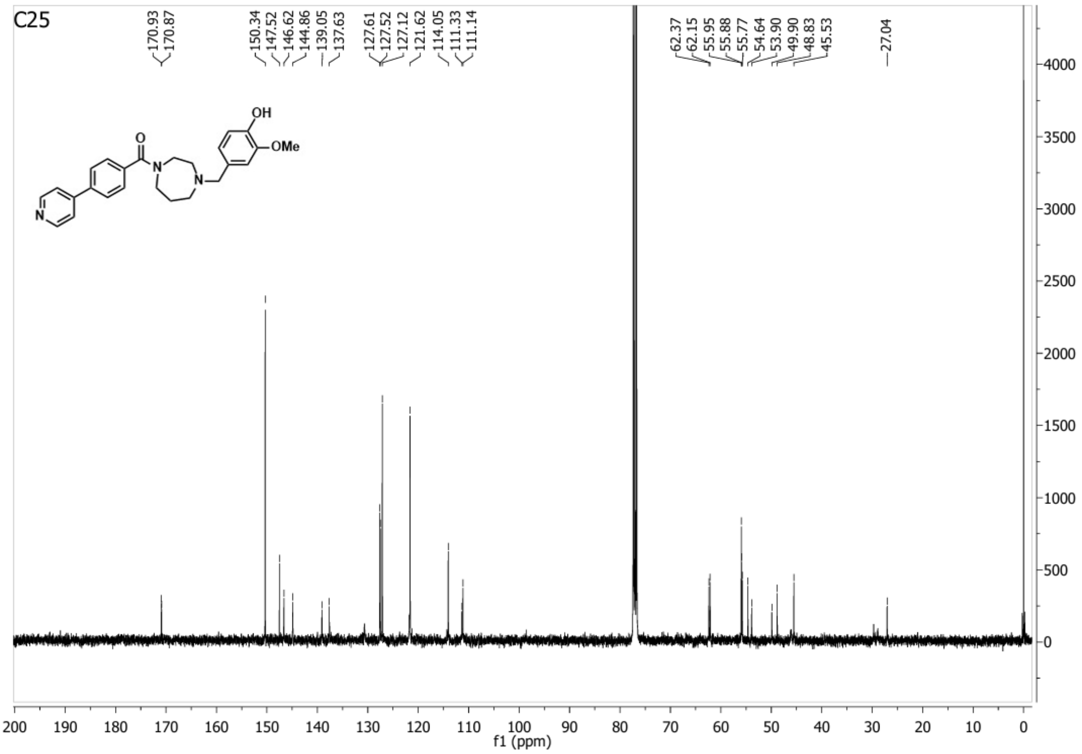

Figure S27. ^{13}C NMR Spectra of C-25

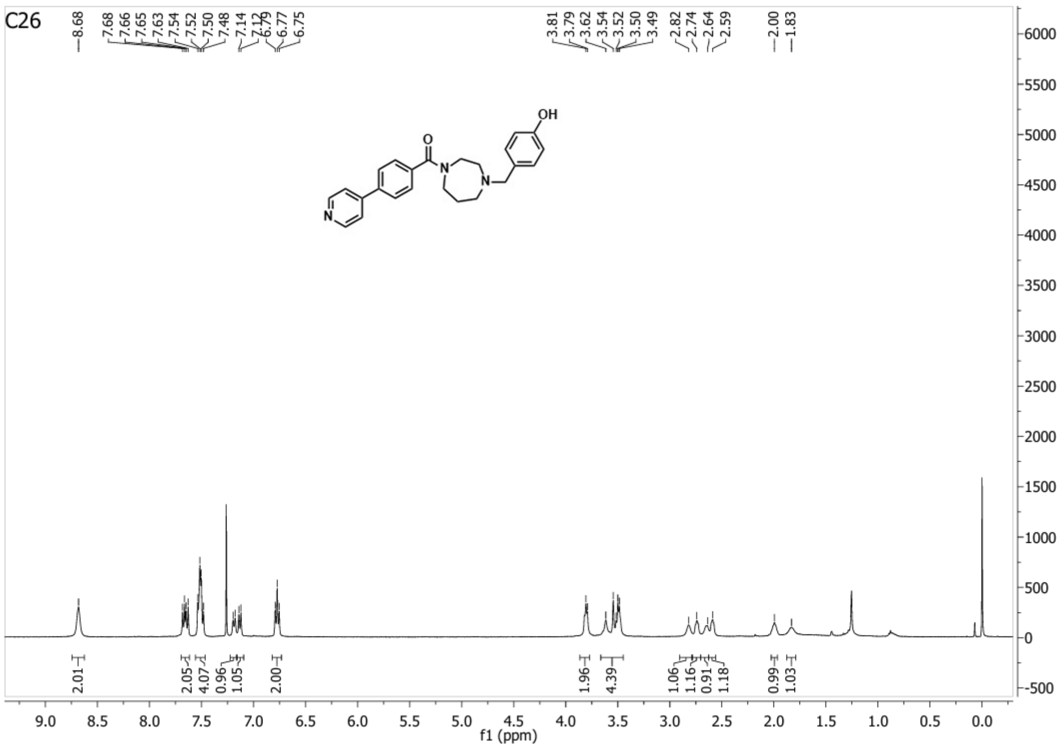

Figure S28. ^{1}H NMR Spectra of C-26

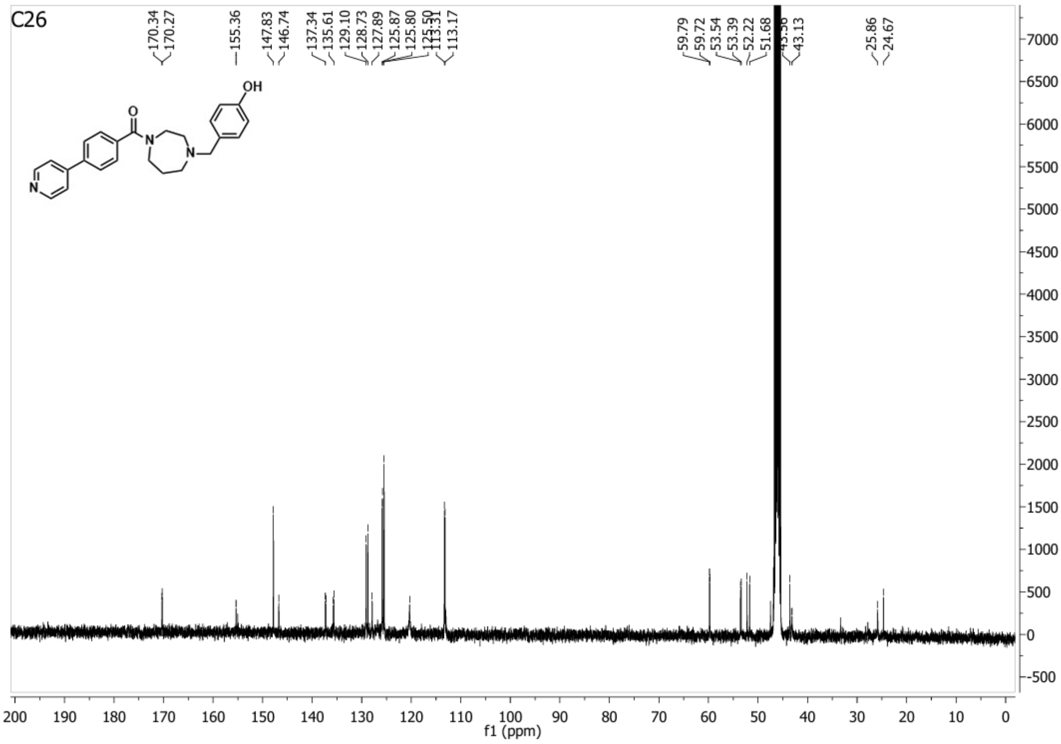

Figure S29. ^{13}C NMR Spectra of C-26

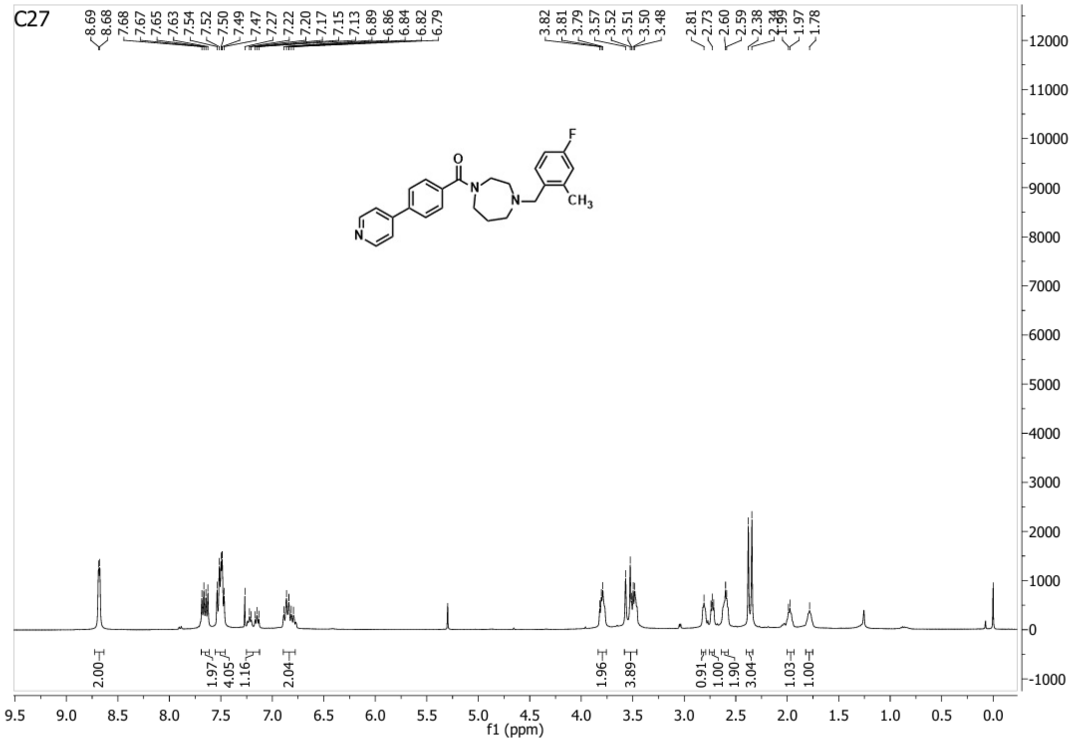

Figure S30. ^{1}H NMR Spectra of C-27

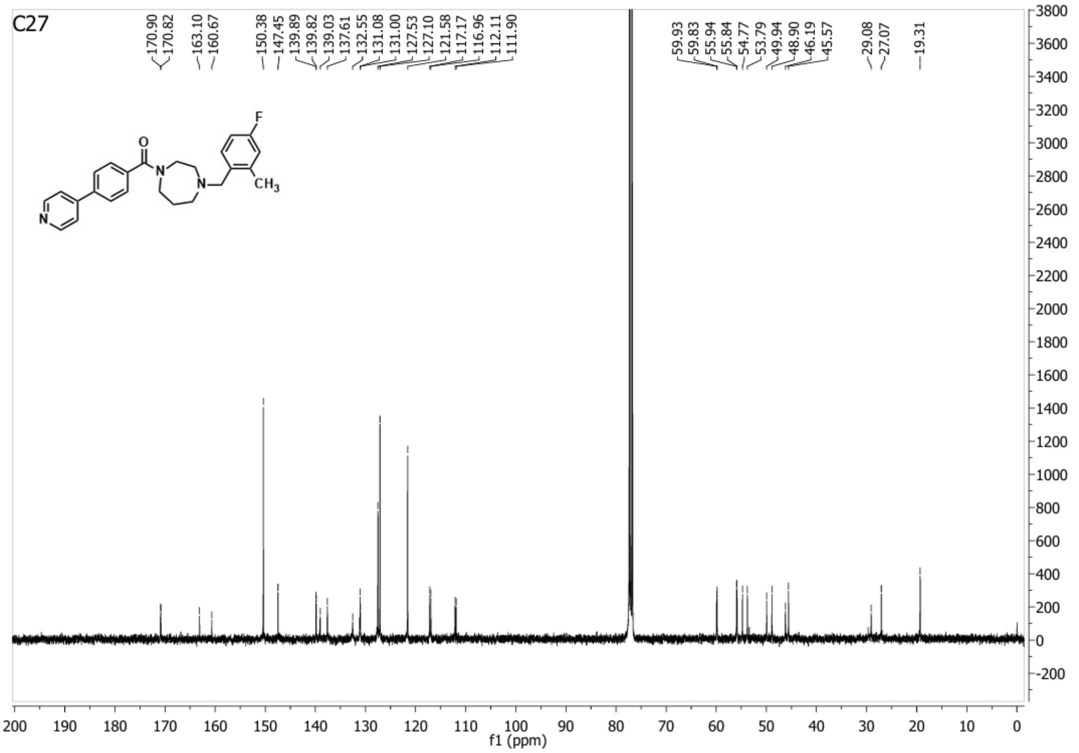

Figure S31. ^{13}C NMR Spectra of C-27

IC$_{50}$=19.10 μM

IC$_{50}$= 35.03 μM
